# Impact of surgery associated with radiotherapy on the prognosis of breast cancer – Guinea Breast Cancer Cohort Study

**DOI:** 10.1002/cnr2.1554

**Published:** 2021-09-22

**Authors:** Bangaly Traore, Mamady Keita, Abdoulaye Toure, Ibrahima Camara, Assiatou Barry, Moussa Koulibaly

**Affiliations:** ^1^ Surgical Oncology Unit of Donka Faculty of Health Sciences and Technics, University Gamal Abdel Nasser of Conakry Guinea; ^2^ Epidemiology Center for Research and Training in infectiology of Guinea (CERFIG) Guinea; ^3^ Laboratory of Pathology of Donka National Hospital Faculty of Health Sciences and Technics, University Gamal Abdel Nasser of Conakry Guinea

**Keywords:** breast cancer, radiotherapy, recurrence, surgery, survival

## Abstract

**Background:**

In a country where radiotherapy (RT) is not available, advocacy based on the relevance of surgery + adjuvant RT in locoregional control and survival is needed.

**Aim:**

To evaluate the impact of surgery with RT on local control and survival compared to surgery alone in breast cancer (BC).

**Methods and results:**

Between 2007 and 2016, 210 patients with BC were retrospectively reviewed, of which 90 patients underwent surgery with RT (group 1) and 120 patients' surgery (group 2). There were several treatment combinations, including surgery combined with neoadjuvant chemotherapy [ACT], RT, and ACT. The results showed 88 (41.9%) cases of relapse, including 31 (34.4%) (group 1) and 57 (47.5%) (group 2) (*p* = .046). Recurrence occurred after a mean time of 1.5 years in group 1 versus 0.66 years in group 2 (*p =* .006). The 5‐year overall and BC‐specific survivals were 49.5% and 62.5%, respectively. The 5‐year survival was 60.0% (group 1) and 40.0% (group 2) (*p* < .05). In a multivariate analysis by Cox model, we found that the risk of death was 1.90 81 (95% CI [1.17 09–3.0701]) higher in group 2 (*p* = .009022), 1.69 85 (95% CI 1.00087–23.86157) in obese patients and decreased by 0.21 (95% CI [0.129–0.368]) in patients who did not relapse (*p* < .001).

**Conclusion:**

In this study with several combination therapies, we cannot confirm that RT improves mainly locally advanced BC prognosis regardless of systemic treatment. However, we found that the risk of death correlated with the absence of RT, overweight, and risk of recurrence. Consideration of combinations of locoregional and systemic therapies, clinicopathological and biological data could improve the relevance of these results with a large sample size.

## 1 INTRODUCTION

Breast cancer (BC) is the most common malignant tumor in women worldwide. In sub‐Saharan Africa, the incidence of BC ranged from 15 to 53 per 100 000 women, which is lower than that in industrialized countries.^1^ In Guinea, this cancer is one of the most common in women with an age‐standardized ratio incidence of 14.5 new cases per 100 000 and with a mortality rate of 7.9 per 100 000.^2^ BC represents the leading cause of consultation at the Surgical Oncology Unit (SOU) of Donka National Hospital with 26% of all cancers.^3^

The treatment of BC is both locoregional (surgery, radiotherapy [RT]) and systemic (chemotherapy, targeted therapy, and hormone therapy). As other methods, surgical treatment has improved considerably since the first description by Halsted in 1891. Breast surgery has been modernized, and becoming less aggressive (Patey's modified radical mastectomy [MRM], breast‐conserving surgery [BCS])^4^ while being curative.^5^ To date, the major challenge for the third world surgeon is to improve the quality of BC treatment through BCS. The most recent systematic overview indicates that after surgery for early BC, RT reduces the absolute cancer mortality by 5% at 10 years.^6^ Similarly, BCS and RT improve 5‐year survival by 8.6% after RT.^6^ The best survival rates, ranging from 70% to 95.5% were recorded in European countries and the United States of America is related to the early diagnosis and optimal access to the means of treatment.^7^ The increase in its incidence and the decrease in mortality reflect the success of screening programs that led to the early detection of BC and the development of adequate therapies in developed countries. However, in low and middle‐resource countries the survival rates are below 60% due to late discovery and difficulties to access complementary therapies such as RT and systemic treatment.^7^, ^8^, ^9^, ^10^ Although its benefits are well known, RT is not yet available in Guinea, and patients are being evacuated to Senegal and other countries. In a country where RT is not available, advocacy based on the relevance of surgery + adjuvant RT in locoregional control and survival is necessary. In this perspective, we assessed the impact of surgery with RT on the prognosis of BC patients in a hospital with limited resources.

## 2 PATIENTS AND METHODS

This study was conducted at the SOU of Donka National Hospital. It was a retrospective cohort study on patients with BC treated by surgery with or without RT between 2007 and 2016.

### 2.1 Population

From 2007 to 2016, a total of 569 patients with histologically confirmed BC were enrolled. Of these, 210 (38.0%) underwent surgery, among which 90 (42.9%) had adjuvant RT and 120 (57.1%) had no RT. We excluded all patients with operated stage 4 and **non‐operated** BC.

The baseline characteristics collected were age, sex, body mass index, inflammatory aspect, histological type, histopronostic grade of Scarff Bloom Richardson, estrogen and progesterone receptor, human epidermal receptor (Her), and the clinical stage based on 8th edition of union for international cancer control–TNM classification.

### 2.2 Surgical treatment

The surgery delay was defined by the time interval between the date of diagnosis and the date of surgery in months. For stage 1 BC, BCS was performed. In stage 2, neoadjuvant chemotherapy (NACT) was administrated before BCS with/or MRM. In stage 3, MRM was performed after NACT. The MRM applied was a modified Patey's method. NACT protocol included anthracyclines and/or taxanes based drugs. After surgery, ACT was administered.

Common standard chemotherapy regimens (repeated every 3 weeks) were:

- Anthracycline‐based including FAC75 (cyclophosphamide 500 mg/m^2^ day 1 + fluorouracil 500 mg/m^2^ day 1 + doxorubicin 75 mg/m^2^ day 1) or FEC100 (cyclophosphamide 500 mg/m^2^ day 1 + fluorouracil 500 mg/m^2^ day 1 + epirubicin 100 mg/m^2^ day 1) intravenous in 3–4 cycles on the first line.
- And taxane‐based with docetaxel 100 mg/m^2^ day 1 or paclitaxel 80 mg/m^2^ weekly in 3–4 cycles in the second line.

### 2.3 Radiotherapy

Adjuvant RT required for all these patients either after BCS for stage 1–3a or MRM for breast locally advanced cancers. The delay for RT was determined by the time interval between the date of surgery and the date of starting RT. RT was performed in Senegal using Cobalt 60 in 82 (89.1%) patients and in other countries (France, Morocco, Mali, Thailand and Switzerland) using a linear accelerator in 10 (9.9%) patients. Patients received 50–50.4Gy in 25–28 fractions at a single dose fraction of 1.8–2Gy on the remaining breast in the case of BCS. For the tumor bed, an electron boost was administered with a median dose of 10 Gy, in 4–5 fractions of 1.8–2.0 Gy. In the case of MRM, 50–50.4 Gy/25–28 fractions were delivered on the chest wall and 46 Gy in 23 fractions were administered on the supraclavicular area in daily fractions of 1.8–2Gy, 5 days per week. The time from breast surgery to RT was recorded.

Combinations of NACT, surgery, ACT, and RT were analyzed.

### 2.4 Data analysis

Patients were follow‐up until March 11, 2017, the endpoint. An analysis of the baseline characteristics was carried out in both groups. A descriptive analysis was performed with the data, using mean (with range), and proportions. The time to recurrence was determined by the difference between the date of detection of the first signs of relapse and the date of surgery. Recurrence‐free included the absence of clinical, biological (CA15.3 marker) or radiological signs of BC on the chest wall/operated breast, regional lymph node areas or other distant organs. Recurrence was considered local‐regional or distant metastatic.

The overall survival was defined as the time interval from the date of diagnosis to death or the date of the last follow‐up. BC specific survival was defined as the time interval between the date of diagnosis and death due to BC other than from other causes. We analyzed the impact of treatment combinations on relapse, overall survival, and BC‐specific survival.

We used the Kaplan–Meier method to estimate the survival function, with and without stratification in the categorical data. We compared each curve using the Log Rank test with a significance level of **0.05**. To estimate the effect of the covariates on the survival of BC patients, we performed a proportional risk analysis according to the Cox model using the stepwise ascending method. Variables with a *p* < 0.20 were included in the multivariate analysis.

The maximum likelihood test was used to compare successive models. Bilateral ***p*** **values <0.05** were considered to be statistically significant. The proportional hazards hypothesis was tested by plotting the Schoenfeld residuals on the scale according to follow‐up. All analysis was performed using Stata 14 (StataCorp, TX, USA).

## 3 RESULTS

There were 206 (98.1%) women and 4 (1.9%) men. The mean age of patients was 47.5 ± 13.0 years (range, 16–85). The mean of the body mass index was 25.7 ± 4.9 kg/m^2^ (range, 14.9–38.7). Co‐morbidities were found in 50 (23.8%) patients. These comorbidities were hypertension 36 (17.1%) cases, diabetes 13 (6.2%) cases, human immunodeficiency virus infection 6 (2.9%) cases and hepatitis B 2 (1.0%) cases. BC was clinically inflammatory in 53 (25.2%) cases. Invasive ductal carcinoma represented 151 (71.9) cases. Histological grade and molecular subtypes (receptor expression and Her2) were known in 97 (46.2%) and 42 (20.0%) patients, respectively. The clinical‐stage was I in 13 (6.2%), II in 45 (21.4%) and III in 152 (72.4%) cases. The clinicopathological baseline characteristics are detailed in Table 1.

**TABLE 1:** Clinicopathological baseline characteristics of breast cancer patients–breast cancer cohort Guinea

Characteristics	All cases
Age (mean, range)	47.5 (16–85)
Sex *n* (%)	
Male	4 (1.9)
Female	206 (98.1)
Body mass index (mean, range)	25.7 (14.9–38.7)
Comorbidities *n* (%)	
Yes	50 (23.8)
No	160 (76.2)
Inflammatory tumor *n* (%)	
Yes	53 (25.3)
No	157 (74.7)
Histology *n* (%)	
Invasive ductal carcinoma	151 (71.9)
Invasive lobular carcinoma	11 (5.2)
Carcinoma no other specified	35 (16.7)
Sarcoma	1 (0.5)
Other carcinomas	12 (5.7)
Scarff Bloom Richardson (SBR) grade *n* (%)	
SBR I	6 (2.9)
SBR II	65 (31.0)
SBR III	26 (12.4)
Missing	113 (53.8)
Molecular profile *n* (%)	
ER/PR+/Her2−	15 (7.1)
ER/PR+//Her2+	5 (2.4)
Her2+	9 (4.3)
ER/PR−/Her2−	13 (6.3)
Missing	168 (80.0)
Stage *n (%)*	
Stage I	13 (6.2)
Stage II	45 (21.4)
Stage III	152 (72.4)
Type of surgery *n (%)*	
Conservative	25 (11.9%)
Radical	185 (88.1%)
Chemotherapy (CT) *n* (%)	
Yes	205 (97.6)
No	5 (2.4)
Neoadjuvant CT	149 (72.7)
Adjuvant CT	131 (63.9)
Neoadjuvant + adjuvant CT	85 (41.5)
Radiotherapy *n (%)*	
Yes	90 (42.9)
No	120 (57.1)

### 3.1 Surgical treatment

Surgery delay ranged from 0 to 70.8 months with an average of 8.4 months. Of the 210 patients, MRM was performed in 185 (88.1%) cases and BCS in 25 (11.9%) cases. Patients who underwent BCS were stage 1, 2 in 20 cases (80.0%) and stage 3a in five cases (20.0%). For MRM, patients were stage 1, 2 in 38 cases (20.5%), and stage 3 in 156 (79.5%).

A total of 205 patients received chemotherapy including 149 (72.7) neoadjuvant, 131 (63.9) adjuvant and 85 (41.5) both (Table 1). Compared to those who did not receive RT, patients who underwent surgery + RT received NACT in 70 (77.8%) versus 19 (22.2%) patients (*p =* .066), ACT in 52 (57.8%) versus 38 (42.2%) patients (*p =* .252), and both NACT and ACT in 33 (36.7%) versus 57 (63.3%) patients (p = 0.066). Table 2 comparing the first and second‐line chemotherapy regimens used shows no difference in the two groups.

**TABLE 2:** Comparison of chemotherapy regimen in the two group

Chemotherapy regimen	First line		Second line	
	Group 1	Group 2	Group 1	Group 2
FAC75 (cyclophosphamide + fluorouracil + doxorubicin)	43	61	0	0
FAC75 (cyclophosphamide + fluorouracil + epirubicin)	43	37	1	0
Docetaxel	3	5	35	24
Paclitaxel (weekly)	0	2	4	6
Others^a^	1	2	0	1
Total	90	107	40	31
*p* value	0.348		0.265	

^a^ Other: CMF (cyclophosphamide + methotrexate + fluorouracile), DV (doxorubicine + vinorelbine).

### 3.2 Radiotherapy

The median time to RT was 6.0 (±4,8) months. RT was performed in 59 patients (65.6%) who had stage 3, 11 (44.30%) patients who underwent BCS and 79 (65.8%) in those who had MRM (*p* = 1.00).

### 3.3 Combination of therapies

The patients had different combination of therapy; the possibilities were:

- NACT + surgery + RT+ ACT in 33 (15.7%) patients,
- NACT + surgery + RT in 37 (17.6%) patients,
- Surgery + ACT + RT in 19 (9.0%) patients,
- NACT + surgery + ACT in 52 (24.8%),
- NACT + surgery in 27 (12.9%) patients,
- Surgery + ACT in 27 (12.9%) patients,
- Surgery + RT 1 (0.5%) patient,
- And surgery alone 14 (6.7%) patients.

Hormonotherapy was administered to 20 patients, 20 of whom were estrogen receptor positive and 15 were progesterone receptor positive.

### 3.4 Follow‐up

These patients were followed for 649.6 person‐years: 328.4 for those patients in group 1 and 321.2 for those in group 2. During the follow‐up, we found 88 (41.9%) cases of relapses, including 31 (34.4%) in group 1 and 57 (47.5%) in group 2 (*p* = .046). These relapses were locoregional in 41 (19.5%) and distant in 61 (29.1%). Locoregional relapse occurred in 10 (11.1%) in group 1 and 31 (25.8%) in group 2 (*p* = .008). The distant relapse included 21 (23.3%) in group 1 and 27 (22.5%) in group 2 (*p* = 1.00). The median relapse time was 1.3 years (IQR 0.41–1.83). The relapse occurred after a median time of 1.5 (IQR 0.58–2.33) for patients in group 1 and 0.66 (IQR 0.25–1.25) in group 2 (*p =* .006).

**FIGURE 1:**
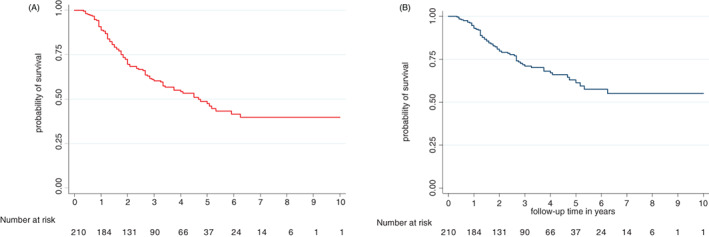
Overall and breast cancer‐specific survivals of patients treated by surgery with/without radiotherapy–breast cancer cohort Guinea

**FIGURE 2:**
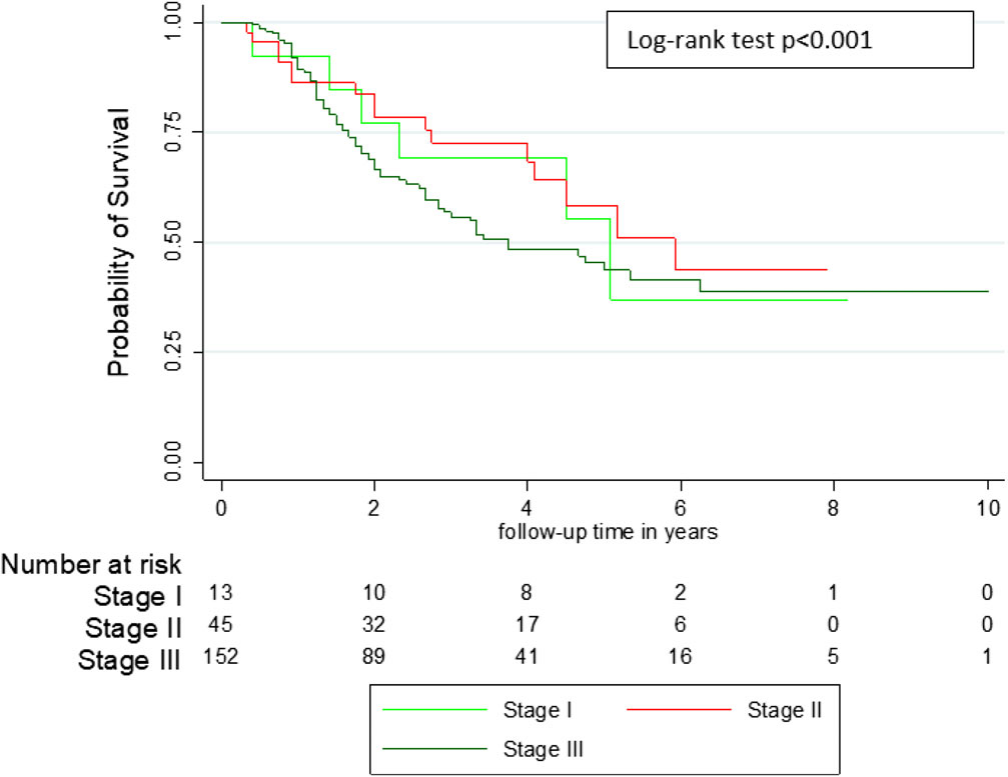
Comparative survival curve of breast cancers according to the clinical stage–Breast cancer cohort Guinea

**FIGURE 3:**
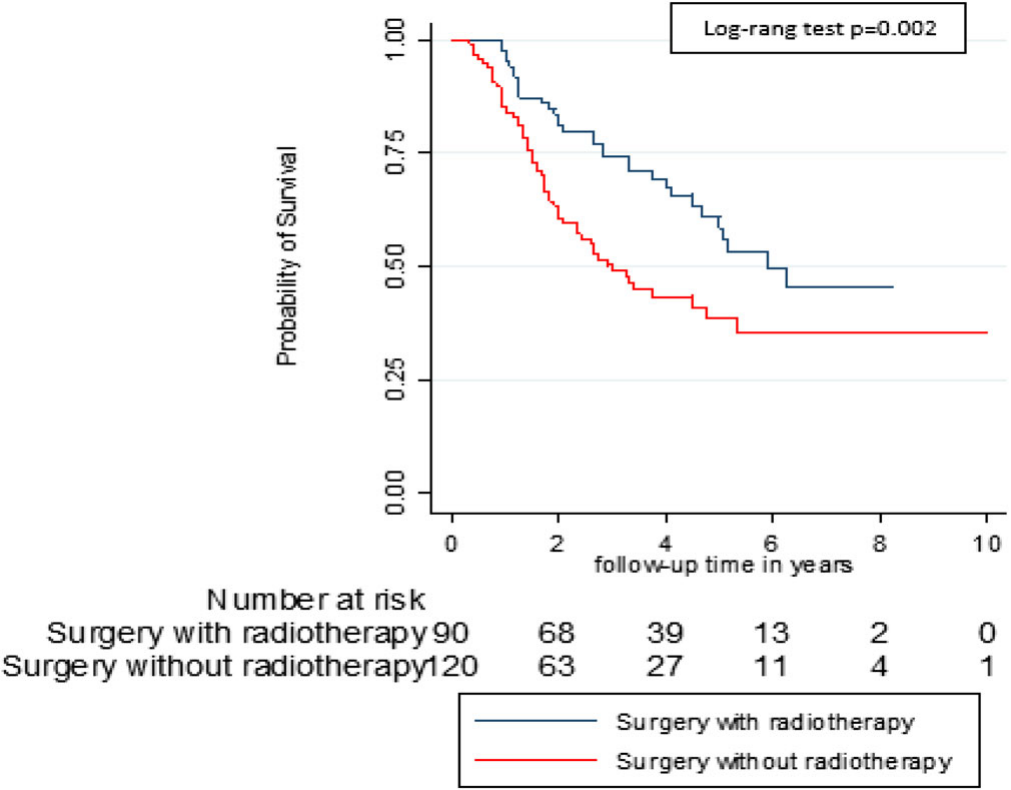
Comparative survival curve of breast cancers treated with and without radiotherapy–breast cancer cohort Guinea

**FIGURE 4:**
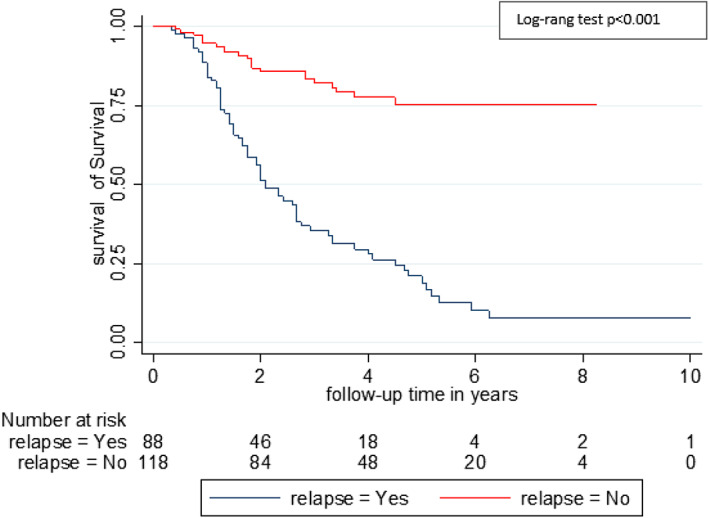
Comparative survival curve according to breast cancer relapse–breast cancer cohort Guinea

A total of 94 (44.8%) patients died including four males and 90 females (*p* = .039). The causes of death were cancer 71 (75.5%), side effects of chemotherapy (SEC) 6 (6.4%), surgical complications (pulmonary embolism) 1 (1.1%) and other 16 (17.2%). The SEC were hematological (neutropenia +/− pancytopenia) (four cases) and heart failure due to cardiomyopathy (two cases). The 16 other causes of death occurring outside the hospital included two traffic accidents, two strokes, and 12 unknowns. For patients with co‐morbidity, the causes of death were for HIV patients: Cancer (six cases); diabetic patients: Cancer (five cases) and hematological SEC (one case); and hypertensive patients: cancer (15 cases), cardiac SEC (two cases), and other causes (three cases). The five‐year overall and BC specific survival were 49.5% and 62.5% respectively (Figure 1). Table 3 presents the univariate analysis of factors associated with patient survival. According to the clinical stage, the 5‐year survival was 56.2% for stage 1, 59.3% for stage 2 and 43.5% for stage 3 (p < 0.0.37) (Figure 2). The 5‐year survival ranged from 40.0% for patients in group 2 to 60.0% for those in group 1 (*p* = .001) (Figure 3). The 5‐year survival in patients with relapsed was lower than those without recurrence (20.8% vs. 75.0%) (*p* < .001) (Figure 4). The Table 4 shows relapse, BC‐specific and overall survival according to treatment combination. Among the treatment combinations, the combination of NACT + Surgery + RT had decreased recurrence rate 9 (10.0%) versus 81 (90.0%) (*p* = .0017); decreased death from all causes 9 (9.6%) versus 85 (90.4%) (*p* = .006) and death from BC 7 (9.9%) versus 64 (90.1%) (*p* = .004). The combination of surgery + RT + ACT decreased death from BC 1 (1.4%) versus 70 (98.6%) (*p* = .004). On the other hand, the death from all causes was lower in patients who received NACT + surgery + ACT 5 (5.3%) versus 89 (94.7%) (*p* = .037). In a multivariate analysis by Cox proportional model, we found that the risk of death was 1.79 (95% CI [1.07–2.97]) higher in group 2 (*p* = .024), 3.45 (95% CI 1.00087–23.86157) in overweight patients, and 4.79 (95% CI [2.86–8.03]) in patients who relapsed (*p* < .001). (Table 5).

**TABLE 3:** Comparison of baseline patient characteristics by status (Living versus death) at last follow‐up date

Characteristics	Patients in live *N* = 117	Patients dead *N* = 103	*p* value
	*n* (% in column)		
Age (median, IQr)	47.0(IQR 36.5–56.0)	57.0 (IQR 40.0–57.0)	0.71
Sex			.002
Male	0 (0)	4 (3.9)	
Female	116(100)	90 (96.1)	
Body mass index (median, IQR)	25.3 (IQR 22.4–28.6)	25.0 (IQR 22.15–28.9)	0.75
Comorbidities			0.404
Yes	24 (20.5)	26 (25.2)	
No	93 (79.5)	77 (74.8)	
Inflammatory tumor *n* (%)			.002
Yes	21 (18.0)	38 (36.9)	
No	96 (82.0)	65 (63.1)	
Histological			0.46
Invasive ductal carcinomas	80(69.0)	71 (75.5)	
Invasive lobular carcinomas	8 (6.9)	3 (3.2)	
Carcinoma no other specified	20 (17.2)	15 (16.0)	
Sarcoma	0 (0.0)	1 (1.1)	
Other carcinomas	8 (6.9)	4 (4.3)	
Scarff Bloom Richardson (SBR) Grade			.06
SBR I	4 (3.5)	2 (2.1)	
SBR II	42 (36.2)	23 (24.5)	
SBR III	10 (8.6)	16 (17.0)	
Missing	60 (51.7)	53 (56.4)	
Molecular profile			0.34
ER/PR + Her2−	10 (8.6)	5 (5.3)	
ER/PR + Her2+	4 (3.6)	1 (1.0)	
Her2+	7 (6.0)	2 (2.1)	
ER/PR‐Her2−	7(6.0)	6 (6.4)	
Missing	88(75.8)	80(85.2)	
Clinical stage			0.37
Stage 1	7 (6.0)	6 (6.4)	
Stage 2	29 (25.0)	16 (17.0)	
Stage 3	80 (69.0)	72 (76.6)	
Chemotherapy			0.584
Yes	108 (92.3)	97 (94.2)	
No	9 (7.7)	6 (5.8)	
Type of surgery			.002
Conservative breast surgery	21 (18.1)	4 (4.3)	
Radical breast surgery	95 (81.9)	90 (95.7)	
Surgery + radiotherapy			.04
Yes	57 (49.1)	33 (35.1)	
No	59 (50.9)	61 (64.9)	
Neoadjuvant chemotherapy			0.222
Yes	78(67.2)	71(75.5)	
No	38(32.8)	23(24.5)	
Adjuvant chemotherapy			0.72
Yes	14(12.1)	19(20.2)	
No	102(87.9)	75(79.8)	
NACT + surgery + RT + ACT			0.107
Yes	14(12.1)	19(19.2)	
No	102(87.9)	75(79.8)	
NACT + surgery + RT			.006
Yes	28(24.1)	9(9.6)	
No	88(75.9)	85(90.4)	
Surgery + RT + ACT			.090
Yes	14(12.1)	5(5.3)	
No	102(87.9)	89(94.7)	
Surgery + CTNA + CTA			.031
Yes	22(19.0)	30(31.9)	
No	94(81.0)	64(68.1)	
NACT + surgery			0.705
Yes	14(7.8)	13(13.8)	
No	102(92.2)	81(86.2)	
Surgery + ACT			0.705
Yes	14(7.8)	13(13.8)	
No	102(92.2)	81(86.2)	
Surgery + RT			0.367
Yes	1(0.9)	0.0(0.0)	
No	115(99.1)	94(100)	
Surgery alone			0.481
Yes	9(7.8)	5(5.3)	
No	107(92.2)	89(94.7)	
Relapse			<.001
Yes	19 (16.2)	69 (73.4)	
No	96 (82.9)	22 (21.4)	
Missing	1(0.9)	3(3.2)	

Abbreviations: ACT, adjuvant chemotherapy; CT, Chemotherapy; ER, estrogen receptor; IQR, interquartile range; NACT, neoadjuvant chemotherapy; PR, progesterone receptor; RT, radiotherapy.

**TABLE 4:** Relapse, breast cancer disease‐specific survival and overall survival according to treatment combination

Treatment combination	Total *n* (%)	Recurrence			BC DSS			OS		
		Yes *n* (%)	No *n* (%)	*p*	Alive *n* (%)	Dead *n* (%)	*p*	Alive *n* (%)	Dead n (%)	*p*	
NACT + S + RT + ACT				0.339			0.160			0.128
Yes	33 (15.7)	17 (18 .9)	16 (13.3)		18 (12.9)	15 (21.1)		14 (12.1)	19 (20.2)	
No	177 (84.3)	73 (81.1)	104 (86.7)		121 (87.1)	56 (78.9)		102 (87.9)	75 (79.8)	
NACT + S + RT				0.017			0.037			0.006
Yes	37 (17.6)	9 (10.0)	28 (23.3)		30 (21.6)	7 (9.9)		28 (24.1)	9 (9.6)	
No	173 (82.4)	81 (90.0)	92 (76.7)		109 (78.4)	64 (90.1)		88 (75.9)	85 (90.4)	
S + RT + ACT				0.150			0.004			0.098
Yes	19 (9.0)	5 (5.6)	14 (11.7)		18 (12.9)	1 (1.4)		14 (12.1)	5 (5.3)	
No	191 (91.0)	85 (94.4)	106 (88.3)		121 (87.1)	70 (98.6)		102 (87.9)	89 (94.7)	
NACT + S + ACT				0.147			0.090			0.037
Yes	52 (24.8)	27 (30.0)	25 (20.8)		29 (20.9)	23 (32.4)		22 (19.0)	30 (31.9)	
No	158 (75.2)	63 (70.0)	95 (79.2)		110 (79.1)	48 (67.6)		94 (81.0)	64 (68.1)	
NACT + S				0.405			0.514			0.836
Yes	27 (12.9)	14 (15.6))	13 (10.8)		16 (11.5)	11 (15.5)		14 (12.1)	13 (13.8)	
No	183 (87.1)	76 (84.4)	107 (89.2)		123 (88.5)	60 (84.5)		102 (87.9)	81 (86.2)	
S + ACT				0.678			1.000			0.836
Yes	27 (12.9)	13 (14.4)	14 (11.7)		18 (12.9)	9 (12.7)		14 (12.1)	13 (13.8)	
No	158 (75.2)	77 (85.6)	106 (88.3)		121 (87.1)	62 (87.3)		102 (87.9)	81 (86.2)	
S + RT				1.000			1.000			1.000
Yes	1 (0.5)	0 (0.0)	1 (0.8)		1 (0.9)	0 (0.0)		1 (0.7)	0 (0.0)	
No	209 (99.5)	90 (100)	119 (99.2)		115 (99.1)	94 (100)		138 (99.3)	94 (100)	
Surgery alone				0.781			1.000			0.584
Yes	14 (6.7)	5 (5.6)	9 (7.5)		9 (6.5))	5 (7.0)		9 (7.8)	5 (5.3)	
No	196 (93.3)	85 (94.4)	111 (92.5)		130 (93.5)	66 (93.0)		107 (92.2)	89 (94.7)	
Total	210 (100)	90 (42.9)	120 (57.1)		139 (66.2)	71 (33.8)		116 (55.2)	94 (44.8)	

Abbreviations: ACT, adjuvant chemotherapy; BC DSS, breast cancer disease specific survival; NA, not applicable; NACT, neoadjuvant chemotherapy; OS, overall survival; p, p value; RT, radiotherapy; S, surgery.

**TABLE 5:** Multivariate Cox regression analysis for factors predicting overall survival, Guinea breast cancer Cohort

Variables	HR	SE	z	*p* > |z|	[IC 95%]
Radiotherapy					
Yes	Ref. [1]				
No	1.79	0.46	2.25	.024	[1.07–2.97]
Relapse					
No	Ref. [1]				
Yes	4.79	1.26	5.96	<.001	[2.86–8.03]
Stage					
Stage 1	Ref. [1]				
Stage 2	1.30	0.89	0.39	0.694	[0.34–5.01]
Stage 3	1.56	0.98	0.71	0.476	[0.45–5.34]
Body mass index					
Normal	Ref. [1]				
Underweight	1.99	1.21	1.12	0.256	[0.59–6.76]
Overweight	3.45	2.19	1.96	.050	[1.0003–11.93]
Moderate obesity	2.10	1.44	1.09	0.276	[0.55–8.06]
Severe obesity	3.54	2.18	1.00	0.277	[0.47–13.73]
Comorbidities					
No	Ref. [1]				
Yes	0.78	0.20	−0.91	0.369	[0.46–1.32]
Type of surgery					
Breast conserving surgery	Ref. [1]				
Modified radical mastectomy	2.22	2.43	1.55	0.121	[0.743–14.18]

Abbreviations: CT, chemotherapy; HR, hazar ratio; *p*, *p* value; Ref, reference; RT, radiotherapy; S, surgery; SE, standard error.

## 4 DISCUSSION

This retrospective cohort study was conducted to access the impact of surgery with or without RT in patients with BC in a country where RT is not available. The limitation was the poor knowledge of molecular subtypes because of the lack of immunohistochemistry in our country. Surgical margins, number of axillary nodes invaded, lymph node invasion, and tumor size were not included in the survival analyses due to many missing data. Also this study highlighted the difficulties of access to both surgical and RT treatment in our country. These difficulties in accessing different treatment methods have already been reported in a previous study, which showed that only 36.0% of patients in our unit were treated.^11^ In another study, Traore et al.^10^ found that only 44.4% of women who had surgery for BC received RT. The limited access to the surgery could be explained by the advanced stage at the diagnosis of BC justifying NACT, then the surgery in case of partial or complete response. This means that patients with advanced stage can only receive surgery if they had NACT as 71.8% of patients in this study. NACT allowed conservative breast surgery in six patients who had T3N0 BC or who have had a tumor over 3 cm in size. The goal of NACT is twofold. First, NACT helps to induce a downstage in the primary tumor volume. Second, it destroys sub‐clinical metastases. However, it should be noted that all BC patients operated on in this study should receive RT. We did not find significant differences in age, gender, co‐morbidities, histological and molecular subtypes between the two groups. In contrast, patients in group 1 were more overweight, had less inflammatory disease, less aggressive, and less advanced BC. Except in six patients with T3N0 BC (stage 3a), all other with locally advanced BC underwent MRM after NACT. While more than three‐quarters of BCS concerned stage 1 and 2 BC. BCS, which is at its beginning in our context is increasingly indicated in highly selected cases of locally advanced BC.^12^, ^13^ However, surgery remains the main means of treatment available in Sub‐Saharan African countries.^5^ While BCS is used to treat more than half of women with BC in the Western countries,^14^, ^15^ surgical treatment remains dominated by radical mastectomy in Africa.^5^ Out of 210 BC patients received in two university hospitals in Bamako (Mali), Togo et al. reported that 68.7% of patients had surgery. Few countries in Sub‐Saharan Africa have RT. In 2013, a study conducted in 14 countries members of the African organization for training and research on cancer (AORTIC) showed that only 36% of countries members possess RT.^16^ In our current study, to receive RT, most patients were evacuated to the neighboring countries where the machines were frequently broken down.

This study showed that more than 89% of RT was performed with telecobalt in Senegal. The mean time to RT was highly variable from one patient to another with an average time of 6 months, while the recommended time after surgery is 3–4 weeks, the time required for healing.^17^ This meantime to RT was much longer than that time in western African countries where RT is available, which range was 2.8–5.6 months.^18^, ^19^ Stefoski Mikeljevic et al.^20^ showed that the delay of RT can influence the oncological outcome of BC. In our context, the delay of RT was related to the lack of RT in our country. Also patients took enough time to find financial resources before going for RT.

The recurrence rate seems higher in this study than that of our previous study^10^ in the same department, 42.7% versus 33.6%. This difference could be related to patient follow‐up problems. The high recurrence rate could also be related to the advanced stage and the lack of adjuvant RT. Due to the small size of our sample, and the lack of case–control studies, we cannot affirm any difference between the recurrence rate after BCS and MRM. Nevertheless, studies have shown that with the same stage, the results of treatments combining surgery and adjuvant RT on locoregional control are equivalent to those of radical surgery for early stages BC.^21^

The locoregional recurrence rate was lower in group 1 than in group 2. RT improves locoregional control by reducing the relapse rate by three times compared to no RT after BCS..^22^, ^23^ Our patients relapsed more rapidly with a median relapse time of 15.6 months compared to 26 months in the study by Dunst et al.^24^ and 27 months according to Siponen.^25^ However, the occurrence of recurrence in our series was influenced by RT; 18.0 months in group 1 versus 7.9 months in group 2.

Most of our patients died from their BC. Other causes of death, including chemotherapy, chemotherapy and co‐morbidities could be prevented. Unknown causes of death need to be explored, especially as we are in a country where infectious diseases are the leading cause of death. The 5‐year overall survival of 49.5% in this study was slightly lower than that of Galukande et al. in Uganda, which was 51.8%..^26^ But BC‐specific survival is nearly 10 years higher. The 5‐year survival rate was significantly better in group 1 than in group 2, with 60.0% and 40.0%, respectively. Previous studies in our unit and in other countries have clearly shown that advanced stage, the lack of RT, and relapse were independent prognostic factors that were associated with a decrease of survival in BC patients.^7^, ^10^, ^26^, ^27^ In addition to the benefice of reduction in mortality, RT associated with conservative surgery reduces the risk of distant recurrence in early‐stage cancers.^28^ Analysis of the impact of treatment combinations showed that radiation therapy decreases the recurrence rate and improves survival when used in combination with neoadjuvant or ACT. Inclusion of locoregional (surgery, RT) and systemic (NACT or ACT) therapies with a large sample size would allow for better evaluation of the impact of RT on survival in these advanced BCs.

## 5 CONCLUSION

In this study with several combination therapies, we cannot confirm that RT improves mainly locally advanced BC prognosis regardless of systemic treatment. However, we found that the risk of death correlated with the absence of RT, overweight, and risk of recurrence.

Consideration of combinations of locoregional and systemic therapies, clinicopathological and biological data could improve the relevance of these results with a large sample size.

## CONFLICT OF INTEREST

T B, K M, T A, C I, B A, and K M received no funding for this publication. The authors have stated explicitly that there are no conflicts of interest in connection with this article.

## AUTHOR CONTRIBUTIONS

**Bangaly, MD, Prof Traore:** Conceptualization (equal); data curation (equal); investigation (equal); methodology (equal); resources (equal); software (equal); supervision (equal); validation (equal); visualization (equal); writing – original draft (equal); writing – review and editing (equal). **Mamady Keita:** Conceptualization (equal); formal analysis (equal); methodology (equal); validation (equal); writing – original draft (equal); writing – review and editing (equal). **Abdoulaye Toure:** Conceptualization (equal); methodology (equal); software (equal); validation (equal); visualization (equal); writing – original draft (equal); writing – review and editing (equal). **Ibrahima Camara:** Data curation (equal); methodology (equal); software (equal); validation (equal); visualization (equal); writing – original draft (equal); writing – review and editing (equal). **Assiatou Barry:** Conceptualization (equal); data curation (equal); formal analysis (equal); methodology (equal); software (equal); validation (equal); visualization (equal); writing – original draft (equal); writing – review and editing (equal). **Moussa Koulibaly:** Conceptualization (equal); formal analysis (equal); methodology (equal); software (equal); validation (equal); visualization (equal); writing – original draft (equal); writing – review and editing (equal).

## ETHICS STATEMENT

In this retrospective study, data were collected anonymously and confidentially. Patients signed the consent form for the use of data contained in their records and we obtained ethical approvement (L005/CNERS/21).

## Data Availability

The data that support the findings of this study are available from the corresponding author upon reasonable request.
